# Marimastat in recurrent colorectal cancer: exploratory evaluation of biological activity by measurement of carcinoembryonic antigen

**DOI:** 10.1038/sj.bjc.6690079

**Published:** 1999-02

**Authors:** J N Primrose, H Bleiberg, F Daniel, S Van Belle, J L Mansi, M Seymour, P W Johnson, J P Neoptolemos, M Baillet, K Barker, A Berrington, P D Brown, A W Millar, K P Lynch

**Affiliations:** University Surgery Unit, Southampton General Hospital, Tremona Rd, Southampton, SO16 6YD, UK; Institut Jules Bordet, Rue Heger-Bordet 1, 1000 Brussels, Belgium; Department of Radiotherapy, Plymouth Hospital NHS Trust, Freedom Fields, Plymouth, PL3 7JJ, UK; Oncologisch Centrum, Universitair Ziekenhuis Gent, De Pintelaan, 9000 Gent, Belgium; Department of Medical Oncology, St George's Hospital, Blackshaw Rd, London, SW17 0QT, UK; ICRF Cancer Medicine Research Unit, Cookridge and St James' University Hospitals, Beckett St, Leeds, LS9 7TF, UK; Department of Surgery, Queen Elizabeth Hospital, Edgbaston, Birmingham, B15 2TH, UK; British Biotech Pharmaceuticals, Watlington Rd, Oxford, OX4 5LY, UK

**Keywords:** marimastat, metalloproteinase inhibitors, colorectal cancer, carcinoembryonic antigen

## Abstract

Marimastat is a specific inhibitor of matrix metalloproteinases that has been shown to be effective in cancer models. A pilot, escalating-dose study of oral marimastat was performed in patients with recurrent colorectal cancer, in whom evaluation of serological response was made by measurement of carcinoembryonic antigen (CEA) levels. The study assessed the safety and tolerability of 4 weeks administration of marimastat, and determined a dose range producing detectable serological effects. Patients were recruited with a serum CEA level greater than 5 ng ml^−1^, and rising by more than 25% over a 4-week screening period. Patients were treated for 28 days and entered into a continuation protocol if a serological response or clinical benefit was observed. Pharmacokinetic and safety data determined that groups of patients were recruited sequentially at 25 mg and 50 mg twice daily, and, thereafter, 10 mg twice daily, 10 mg once daily, 5 mg once daily and 20 mg once daily. A biological effect (BE) was defined as a CEA value on day 28 no greater than on day 0; a partial biological effect (PBE) was defined as a rise in CEA over the 28-day treatment period of less than 25%. Of 70 patients recruited, 63 completed the 28-day treatment period, and 55 were eligible for cancer antigen analysis. Examination of the dose–effect relationships provides evidence for a causal relationship between marimastat and biological effects: the proportion of patients with BE or PBE was higher with twice daily dosing (16 out of 25, 64%) than with once daily dosing (11 out of 30, 37%) (*P* = 0.043, χ^2^ test). Furthermore, the median rates of rise of CEA fell markedly during treatment compared with the screening period for patients receiving twice daily marimastat (*P* < 0.0001), but not for patients receiving marimastat once daily (*P* = 0.25). Musculoskeletal adverse events emerged as the principal drug-related toxicity of marimastat, occurring in a dose- and time-dependent fashion. It was concluded that marimastat was associated with dose-dependent biological effects in cancer patients. The occurrence of musculoskeletal side-effects define 25 mg twice daily as the upper limit of the dose range for continuous use in further studies. Therefore, a dose range of 20 mg once daily to 25 mg twice daily seems appropriate for further studies, which should aim to demonstrate the efficacy of the drug in terms of conventional clinical end points and describe the long-term tolerability of this novel agent. © 1999 Cancer Research Campaign


*British Journal of Cancer* (1999) **79**(3/4), 509–514
© 1999 Cancer Research Campaign
Article no. bjoc.1998.0079

### Marimastat in recurrent colorectal cancer: exploratory evaluation of biological activity by measurement of carcinoembryonic antigen

**JN Primrose[1], H Bleiberg[2], F Daniel[3], S Van Belle[4], JL Mansi[5], M Seymour[6], PW Johnson[6], JP Neoptolemos[7], M Baillet[8], K Barker[8], A Berrington[8], PD Brown[8], AW Millar[8] and KP Lynch[8]**

[1]University Surgery Unit, Southampton General Hospital, Tremona Rd, Southampton SO16 6YD, UK; [2]Institut Jules Bordet, Rue Heger-Bordet 1, 1000 Brussels, Belgium; [3]Department of Radiotherapy, Plymouth Hospital NHS Trust, Freedom Fields, Plymouth PL3 7JJ, UK; [4]Oncologisch Centrum, Universitair Ziekenhuis Gent, De Pintelaan, 9000 Gent, Belgium; [5]Department of Medical Oncology, St George's Hospital, Blackshaw Rd, London SW17 0QT, UK; [6]ICRF Cancer Medicine Research Unit, Cookridge and St James' University Hospitals, Beckett St, Leeds LS9 7TF, UK; [7]Department of Surgery, Queen Elizabeth Hospital, Edgbaston, Birmingham B15 2TH, UK; [8]British Biotech Pharmaceuticals, Watlington Rd, Oxford OX4 5LY, UK

**Summary** Marimastat is a specific inhibitor of matrix metalloproteinases that has been shown to be effective in cancer models. A pilot, escalating-dose study of oral marimastat was performed in patients with recurrent colorectal cancer, in whom evaluation of serological response was made by measurement of carcinoembryonic antigen (CEA) levels. The study assessed the safety and tolerability of 4 weeks administration of marimastat, and determined a dose range producing detectable serological effects. Patients were recruited with a serum CEA level greater than 5 ng $ml^{-1}$, and rising by more than 25% over a 4-week screening period. Patients were treated for 28 days and entered into a continuation protocol if a serological response or clinical benefit was observed. Pharmacokinetic and safety data determined that groups of patients were recruited sequentially at 25 mg and 50 mg twice daily, and, thereafter, 10 mg twice daily, 10 mg once daily, 5 mg once daily and 20 mg once daily. A biological effect (BE) was defined as a CEA value on day 28 no greater than on day 0; a partial biological effect (PBE) was defined as a rise in CEA over the 28-day treatment period of less than 25%. Of 70 patients recruited, 63 completed the 28-day treatment period, and 55 were eligible for cancer antigen analysis. Examination of the dose–effect relationships provides evidence for a causal relationship between marimastat and biological effects: the proportion of patients with BE or PBE was higher with twice daily dosing (16 out of 25, 64%) than with once daily dosing (11 out of 30, 37%) ($P = 0.043$, $\chi^2$ test). Furthermore, the median rates of rise of CEA fell markedly during treatment compared with the screening period for patients receiving twice daily marimastat ($P < 0.0001$), but not for patients receiving marimastat once daily ($P = 0.25$). Musculoskeletal adverse events emerged as the principal drug-related toxicity of marimastat, occurring in a dose- and time-dependent fashion. It was concluded that marimastat was associated with dose-dependent biological effects in cancer patients. The occurrence of musculoskeletal side-effects define 25 mg twice daily as the upper limit of the dose range for continuous use in further studies. Therefore, a dose range of 20 mg once daily to 25 mg twice daily seems appropriate for further studies, which should aim to demonstrate the efficacy of the drug in terms of conventional clinical end points and describe the long-term tolerability of this novel agent.

**Keywords**: marimastat; metalloproteinase inhibitors; colorectal cancer; carcinoembryonic antigen

Matrix metalloproteinases (MMPs) are a group of enzymes responsible for the degradation of extracellular matrix that occurs during tissue formation and remodelling (Matrisian, 1990). In health, the activity of MMPs is regulated at several levels, including their secretion as latent proenzymes and inhibition of their active form by endogenous tissue inhibitor of metalloproteinases (TIMPs) (Kleiner and Stetler-Stevenson, 1993). It is now recognized, however, that unregulated and excessive MMP activity explains many of the behavioural features of cancer (Liotta and Stetler-Stevenson, 1990), rheumatoid arthritis (Gordon et al, 1993), osteoarthritis (O'Byrne et al, 1995), inflammatory bowel disease (Saarialho-Kere et al, 1996), neurodegenerative diseases (Gijbels et al, 1992), and cerebral haemorrhage (Rosenberg, 1995). Therefore, the inhibition of MMPs is a potential strategy for the development of novel treatments for these diseases.

*Received 20 January 1998*
*Revised 10 June 1998*
*Accepted 15 June 1998*

*Correspondence to:* JN Primrose, University Surgery Unit, Southampton General Hospital, Southampton SO16 6YD, UK

Screening of many synthetic MMP inhibitors in the early 1990s identified marimastat as the first such compound to show good absorption after oral administration to animals (Beckett et al, 1996). Subsequent administration to healthy volunteers suggested a rapidly absorbed and well-tolerated drug, with an estimated terminal elimination half-life of 8–10 h (Millar et al, 1998). Marimastat is a potent and reversible inhibitor of MMPs, exhibiting $IC_{50}$s in the nanomolar range against interstitial collagenase (MMP-1), gelatinase A (MMP-2), gelatinase B (MMP-9), matrilysin (MMP-7), stromelysin 1 (MMP-3) and metalloelastase (MMP-12). It has little or no activity against unrelated metalloproteinases such as enkephalinase. Studies in animal cancer models with marimastat and its predecessor, batimastat, indicated that these compounds could successfully inhibit tumour growth and metastasis (Brown and Giavazzi, 1995).

The potential action of marimastat in cancer is cytostatic by inhibiting the breakdown of the extracellular matrix, thereby causing the restriction of tumour growth. It is not cytotoxic and would not be expected to cause reduction in tumour size in human studies. As the drug's actions were anticipated to be subtle, it was thought that trial design employing conventional radiological, clinical or serological measurements of tumour response or progression might fail to detect clinically important activity. An alternative might be trials assessing 'response' in terms of maintenance of stable disease. However, it was not considered suitable initially to treat patients for longer than 28 days with this new class of drug. The relevance of disease stability over such a short period is questionable. Therefore, a novel approach to the conduct of phase I and II trials was undertaken in the early development of marimastat.

It was proposed that changes in the levels of the glycoprotein carcinoembryonic antigen (CEA) could be employed as a surrogate marker for the biological activity of marimastat in patients with colorectal cancer. CEA has been used in the clinical management of patients with colorectal cancer for more than three decades (Gold and Freedman, 1965), and has found roles in the detection of recurrent disease (Minton et al, 1985; Quentmeier et al, 1990; Hida et al, 1996) and the monitoring of response to chemotherapy in patients with advanced cancer (Allen-Mersh et al, 1987; Ward et al, 1993). Correlations have been observed between the rate of rise of CEA and reduced survival, and between falls in CEA and improved survival, in patients being treated for colorectal cancer (Sugarbaker et al, 1976; Allen-Mersh et al, 1987; Nakayama et al, 1997). These correlations are not obscured by substantial interpatient variability in baseline CEA concentrations (Allen-Mersh et al, 1987).

Expression of many of the MMPs has been reported in colorectal cancer (Levy et al, 1991; Gray et al, 1993; Zucker et al, 1993; Newell et al, 1994); increased expression of gelatinase B (MMP-9) has been implicated as an independent predictor of recurrence and outcome (Zeng et al, 1996), and expression of matrilysin (MMP-7) has been correlated with disease progression (Ishikawa, 1996). Moreover, MMP inhibitors have been shown to be effective in reducing tumour growth and spread in several xenograft models of human colorectal cancer (Wang et al, 1994; Watson et al 1995; An et al, 1997).

On the basis of these observations, colorectal cancer was chosen as one of the target cancers to be assessed in the marimastat early trial programme, some of the results of which have now been published (Nemunaitis et al, 1998). By examining the effect of marimastat upon the rate of rise of serum CEA, this study aimed to explore the relationship between marimastat dosing and detectable biological effects. In addition, it aimed to evaluate the tolerability of a range of doses and schedules of marimastat in patients with advanced colorectal cancer. The study was sponsored by British Biotech Pharmaceuticals, Oxford, UK, who supplied the drug.

### PATIENTS AND METHODS

### Patients

Patients with previously resected histologically proven colorectal cancer were selected for this study on the basis of a level of CEA above 5 ng ml$^{-1}$, and rising by 25% or more over a 4-week period, before study entry. An Eastern Cooperative Oncology Group (ECOG) performance status of 0–2 and a predicted survival of 3 months or more was required. Patients may have received prior chemotherapy, but were excluded if (i) surgery had been performed in the previous month; (ii) bilirubin and liver enzymes were greater than three times, and creatinine greater than twice, the upper limit of normal; (iii) albumin was less than 25 gl$^{-1}$; or (iv) if there was evidence of weight loss greater than 10% in the previous 3 months.

Patients were recruited into the study from seven centres, five in the UK and two in Belgium. The protocol and protocol amendments were reviewed by the research ethics committee at each investigational centre and approved. All patients provided witnessed written informed consent to participate in the study, and the study was conducted in accordance with the European Guidelines on Good Clinical Practice.

### Objectives

This study aimed to investigate the effect of marimastat on the rate of rise of CEA and to define the safety and tolerability of 4 weeks administration of the drug. Together with the assessment of the pharmacokinetic profiles of marimastat, these measures were to determine an effective dose range, with adequate tolerability for longer term studies.

### Treatment

Gel capsules containing 5, 10, 25 or 50 mg of marimastat were provided by British Biotech Pharmaceuticals, Oxford, UK. Patients received their first dose of marimastat after satisfactory completion of screening procedures at –5 and –1 weeks. It was originally intended in this open pilot dose-escalation study that sequential groups of ten patients would receive doses of 25, 50 and 100 mg twice daily for a period of 28 days. The starting dose of 25 mg twice daily was decided on the basis of data from healthy volunteer studies (Millar et al, 1998), and escalation beyond each dose level was dependent on satisfactory safety data received from patients treated up to that time. Each group was to complete recruitment before the study progressed to the next dose. In the event, pharmacokinetic and safety data ruled out escalation beyond the 50 mg dose, and dose de-escalation occurred, with sequential groups of patients recruited at 10 mg twice daily, 10 mg once daily, 5 mg once daily and 20 mg once daily.

Continued administration of marimastat was allowed beyond 28 days in patients who were considered to be responding to therapy as defined by the response criteria below, or when in the opinion of the investigator the patient was benefiting from the drug. Dose reduction was possible when patients experienced marimastat-related toxicity of less than grade 3.

### End points

After screening, samples were taken for the measurement of CEA at days 0, 7, 14 and 28. CEA measurements were made at local laboratories with each patient's results being measured by the same laboratory. Inter-laboratory variability was not anticipated to confound results because rates of rise of CEA levels rather than absolute values were used for analysis of results. Criteria for response were defined as follows: a biological effect was defined as a rate of rise of CEA during the 28-day marimastat treatment period ≤0%; a partial biological effect was defined as a rate of rise of CEA during marimastat treatment >0% and ≤25%; non-responders were defined as those patients displaying a rate of rise of CEA

 © Cancer Research Campaign 1999

during marimastat treatment of >25%, or who withdrew because of disease progression, or who died within 6 weeks of starting treatment. In the absence of evaluable screening or treatment data, patients were considered to be non-evaluable.

Because samples were not always taken at scheduled times, a computerized preanalysis algorithm was devised which selected the most appropriate antigen measurements for analysis in an unbiased manner (see below). In these analyses, 48 out of 55 (87%) evaluable patients had the required screening antigen measurements recorded within 6 weeks before entry, and 45 out of 55 (82%) had treatment antigen values recorded at the precise protocolized times.

After review of the results of changes in CEA levels, analysis was also made of the effect of marimastat on lactate dehydrogenase (LDH). This enzyme is a marker of cellular turnover and destruction and is biochemically unrelated to cancer antigen expression. LDH levels have been used to guide prognosis in patients with cancer, and have been shown to be associated with time to disease progression and predictive of survival in colorectal cancer (Schwartz, 1992; Fountzilas et al, 1996).

Blood samples were taken for pharmacokinetic evaluation on days 0 (0 and 2 h), 14 and 28. Further samples were taken in patients receiving marimastat for more than 28 days. Clinical chemistry and haematology, urinalysis and vital signs were monitored throughout the study. All adverse events were recorded, whether thought related to marimastat or not.

### Application of algorithm for tumour marker measurement

The computerized algorithm was derived from the study protocol and stipulated that patients were to have two screening (S1 and S2) and two treatment (T1 and T2) CEA values determined at the same laboratory. S1 was to be measured more than 2 weeks before S2, but less than 13 weeks before the start of the study. S2 must have been taken within 4 weeks of the start of treatment, and be greater than 5 ng $ml^{-1}$. The rate of rise of CEA averaged over 28 days must have been ≥25%. T1 was required to be measured less than 7 days before, and less than 2 days after the start of treatment. T2 was to be measured between 22 and 34 days after the start of treatment, but no more than 2 days after the discontinuation of treatment. All patients who failed to meet one or more of these inclusion criteria were to be excluded from the primary analyses.

### Statistical analysis

Categorical data were analysed using the $\chi^2$ test. Differences between patient groups at baseline were analysed using Kruskal–Wallis and Wilcoxon tests. Continuous data were analysed by the Wilcoxon signed rank test and Wilcoxon rank sum test. Before results were analysed, a comparison of patients receiving once daily doses and patients receiving twice daily doses was planned.

### RESULTS

### Patient population

A total of 70 patients were recruited, of whom seven did not complete the 28-day treatment period; five of these seven were evaluable for toxicity and two failed to attend. Three of the five discontinued because of adverse events, one patient died and one experienced disease progression. Of 63 patients completing the first part of the study, 31 continued receiving marimastat for periods of up to 316 days.

Patient characteristics are listed in Table 1 including data by dose group. Colorectal adenocarcinoma was confirmed in 65 patients; the remaining five patients were recorded as having colorectal cancer of unconfirmed histological type. All but two patients had undergone surgery for their cancer, and most had received chemotherapy, predominantly for treatment of recurrent disease. At the time of entry into the study, 68 patients had radiological evidence of visceral metastatic disease, one had lymphatic metastases, and one had regional disease only. Patients were recruited in approximately equal numbers to each of the dose groups. No baseline characteristics were significantly different between dose groups, or between patients treated by once daily or twice daily doses.

**Table 1** Patient characteristics

| Characteristics | Total | 5 mg once daily | 10 mg once daily | 20 mg once daily | 10 mg twice daily | 25 mg twice daily | 50 mg twice daily |
|---|---|---|---|---|---|---|---|
| No. of patients | 70 | 11 | 12 | 14 | 11 | 11 | 11 |
| No. with evaluable CEA data | 55 | 9 | 9 | 12 | 8 | 9 | 8 |
| No. continuing after day 28 | 31 | 6 | 1 | 9 | 6 | 3 | 6 |
| Age | | | | | | | |
| Mean | 63 | 64 | 63 | 62 | 62 | 60 | 65 |
| Range | 40–87 | 45–81 | 47–75 | 46–87 | 50–81 | 40–74 | 59–71 |
| Stage at first presentation | | | | | | | |
| II | 11 | 1 | 2 | 3 | 2 | 2 | 1 |
| III | 42 | 10 | 6 | 7 | 7 | 5 | 7 |
| IV | 17 | 0 | 4 | 3 | 2 | 4 | 3 |
| Screening rate of rise of CEA % | 56 | 60 | 59 | 61 | 57 | 91 | 35 |
| Albumin $gl^{-1}$ | 38 | 35 | 36 | 36 | 42 | 36 | 40 |
| LDH $Ul^{-1}$ | 596 | 633 | 564 | 616 | 320 | 959 | 658 |

© Cancer Research Campaign 1999

**Table 2** Biological responses observed for patients with evaluable screening and treatment CEA results

| Dose | BE (%) | PBE (%) | NR (%) | Total | NE |
|---|---|---|---|---|---|
| 5 mg once daily | 0 (0) | 3 (33) | 6 (67) | 9 | 2 |
| 10 mg once daily | 0 (0) | 3 (33) | 6 (67) | 9 | 3 |
| 20 mg once daily | 4 (33) | 1 (8) | 7 (58) | 12 | 2 |
| All once daily doses | 4 (13) | 7 (23) | 19 (63) | 30 | 7 |
| 10 mg twice daily | 2 (25) | 4 (50) | 2 (25) | 8 | 3 |
| 25 mg twice daily | 1 (11) | 2 (22) | 6 (67) | 9 | 2 |
| 50 mg twice daily | 6 (75) | 1 (13) | 1 (13) | 8 | 3 |
| All twice daily doses | 9 (36) | 7 (28) | 9 (36) | 25 | 8 |

BE, biological effect; PBE, partial biological effect; NR, non-responder, NE, non-evaluable.

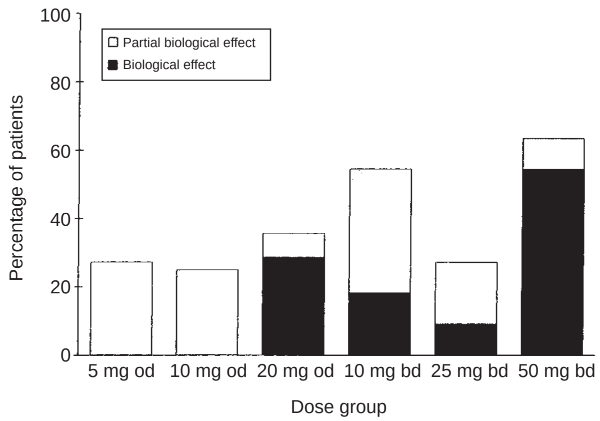

**Figure 1** Percentage biological effect (BE) and partial biological effects (PBE) by dose group, in which BE is defined as a CEA value on day 28 no greater than that on day 0 and PBE is defined as a rise in CEA over the 28-day treatment period of less than 25%

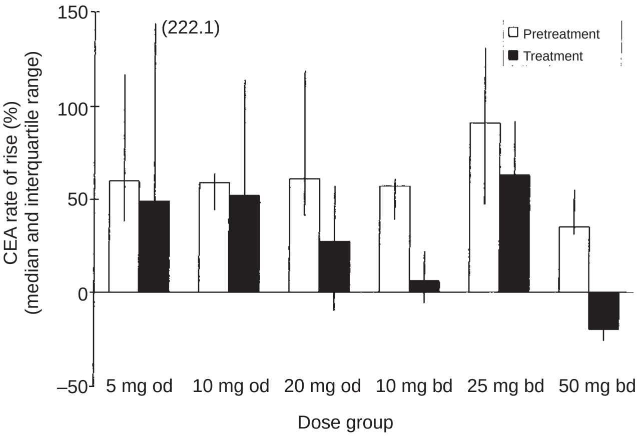

**Figure 2** Median percentage rate of rise of CEA during screening and treatment with marimastat, by dose group with upper and lower quartiles

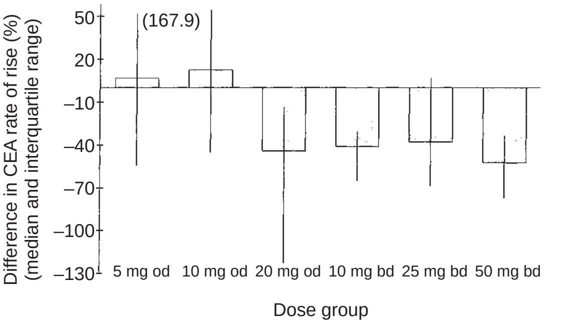

**Figure 3** Median difference between screening and treatment percentage rate of rise of CEA, by dose group with upper and lower quartiles

### Efficacy assessments

In total, 55 (79%) of the 70 patients were eligible for cancer antigen analysis under the rules of the algorithm. The 15 patients excluded from analysis comprised three who failed to complete the study (for reasons other than death or disease progression, this group being categorized as non-responders), 11 who had absent or invalid screening or treatment data, and one whose screening rate of rise was less than 25%.

The results categorized by biological effect (≤0% rise) and partial biological effect (≤25%, >0% rise) are shown by dose group in Table 2 and Figure 1. Only partial biological effects were observed at 5 and 10 mg once daily. Both biological and partial biological effects were observed in the other four dose groups, with the highest combined effects seen at 10 and 50 mg twice daily. The proportion of patients showing a biological or partial biological effect was higher with twice daily than once daily dosing (16 out of 25, 64% compared with 11 out of 30, 37%, $P = 0.043$, $\chi^2$ test). An additional eight patients with screening data ineligible by algorithm but with eligible treatment data were included in an intention to treat analysis. Results were essentially unchanged; the proportion of patients showing a biological or partial biological effect was higher with the twice daily than once daily dosing (20 out of 30, 67% compared with 14 out of 33, 42%, $P = 0.054$, $\chi^2$ test).

Median rates of rise of CEA at screening and during treatment are illustrated in Figure 2. Small falls were apparent in those patients receiving 5 and 10 mg once daily. Changes were more substantial in the higher dose groups, and the difference in those patients receiving twice daily doses of marimastat was more evident ($P < 0.0001$) than in those patients receiving marimastat once daily ($P = 0.25$). The same relationship was observed when the median differences between screen and treatment rates of rise of CEA were charted (Figure 3). The median screening rate of rise of CEA was higher in those patients receiving 25 mg twice daily (91%) than for other groups (35–61%) (Figure 2), and this may explain the relatively low proportion of patients in this group showing biological or partial biological effects (Figure 1).

Trough plasma levels of marimastat are presented by dose in Table 3. For a given dose, trough plasma levels were three- to four-fold higher than that observed in healthy volunteers (Millar et al, 1998). This increase is probably related to pharmacokinetic differences between young healthy males and older patients with advanced malignancy. Considerable intersubject variability was apparent within dose groups, although, with the exception of the group receiving 10 mg once daily, an approximately linear relationship with mean values was observed with increasing dose. Patients were divided into quartiles on the basis of trough plasma levels, and the median difference in treatment and screening percentage rate of rise of CEA for each group was calculated. Median differences for the low (≤23.0 μg $l^{-1}$), low/middle (>23.0

 © Cancer Research Campaign 1999

**Table 3** Trough plasma levels (μg l⁻¹) by dose

| Dose | *n* | Range | Mean (SE) | Median |
|---|---|---|---|---|
| 5 mg once daily | 5 | 8.2–22.7 | 12.6 (2.6) | 11.5 |
| 10 mg once daily | 10 | 10.2–100.9 | 45.2 (10.1) | 47.1 |
| 20 mg once daily | 11 | 8.2–102.4 | 41.5 (9.9) | 25.1 |
| 10 mg twice daily | 11 | 21.5–131.5 | 52.0 (10.5) | 40.6 |
| 25 mg twice daily | 9 | 69.8–314.6 | 165.8 (28.3) | 145.4 |
| 50 mg twice daily | 9 | 138.0–753.4 | 286.2 (67.0) | 182.4 |

*n*, number of patients with recorded trough plasma levels.

**Table 4** Summary of adverse events occurring in 28-day study period (all causalities)

| | Once-daily doses | | | Twice-daily doses | | |
|---|---|---|---|---|---|---|
| | 5 mg | 10 mg | 20 mg | 10 mg | 25 mg | 50 mg |
| Total recruited | 11 | 12 | 14 | 11 | 11 | 11 |
| No. with events | 11 | 12 | 12 | 10 | 11 | 11 |
| Arthralgia | 4 | 2 | 4 | 4 | 0 | 4 |
| Back pain | 1 | 0 | 2 | 2 | 2 | 0 |
| Myalgia | 2 | 2 | 2 | 3 | 3 | 5 |
| Abdominal pain | 5 | 4 | 2 | 2 | 3 | 0 |
| Anorexia | 2 | 1 | 1 | 1 | 4 | 1 |
| Constipation | 4 | 2 | 1 | 1 | 0 | 1 |
| Mouth dry | 0 | 1 | 3 | 0 | 0 | 0 |
| Nausea | 2 | 0 | 2 | 0 | 4 | 0 |
| Dyspnoea | 1 | 1 | 0 | 4 | 0 | 2 |
| Ascites | 0 | 3 | 2 | 0 | 1 | 1 |
| Fatigue | 1 | 2 | 2 | 2 | 0 | 3 |

and ≤63.5 μg l⁻¹), high/middle (>63.5 and ≤138.0 μg l⁻¹), and high (>138.0 μg l⁻¹) quartiles were 35%, 33%, 65% and 52% respectively. Differences between the groups did not reach statistical significance.

A retrospective analysis was performed on the effect of marimastat on LDH. At day 28, the median percentage changes from baseline for the 5 mg once daily, 10 mg once daily, 20 mg once daily, 10 mg twice daily, 25 mg twice daily and 50 mg twice daily groups were +12.4%, +9.0%, –4.4%, –0.7%, –7.8% and –12.4% respectively. The changes in LDH levels were not significantly different when comparing twice and once daily dosing ($P = 0.06$).

### Safety assessments

Sixty-eight patients were assessable for toxicity. Adverse events occurring in at least three patients in one of the dose groups during the study period are presented in Table 4. This table incorporates all reported events, whether thought to be related to marimastat or not. The most commonly reported events were those related to the musculoskeletal and gastrointestinal systems. Four serious events were reported as being possibly related to marimastat, including single cases of fatigue, general body pain, renal dysfunction and jaundice. There were no obvious adverse trends in laboratory values with marimastat treatment, although many out of range values were recorded both before and during treatment.

When considering adverse events reported to be definitely, probably or possibly related to marimastat, it was apparent that the principal toxicity of marimastat reported in this study was the occurrence of reversible musculoskeletal events. Symptoms included myalgia, arthralgia and tendinitis, predominantly of the upper limbs. The times of occurrence of musculoskeletal symptoms requiring dose reduction or withdrawal are described in Table 5. Although musculoskeletal events occurred in a dose- and time-dependent fashion, no clear relationship was observed between the occurrence of musculoskeletal adverse events and trough serum levels of marimastat. Four other patients who completed the 28-day study period suffered musculoskeletal symptoms of at least moderate severity and, possibly for this reason, were not entered into the continuation protocol.

### DISCUSSION

This study has taken a novel approach to the clinical development of a cytostatic agent in attempting to show biological activity by the effect of marimastat on the rates of rise of cancer antigens. It has succeeded in this by showing changes in the rates of rise of CEA in colorectal cancer which were suggestive of a dose-dependent biological effect. These results allow estimation of a dose range suitable for longer term controlled studies, which might hope to establish the clinical efficacy of marimastat.

Biological and partial biological effects were seen most commonly in the patients treated with higher doses of marimastat; a significant increase in response was observed between those patients receiving marimastat twice daily and those receiving marimastat once daily. The dose-dependent effect on rate of rise of CEA was also apparent when assessing the relative falls in rate of rise of CEA between the dose groups. Increased effects on the rate of rise of CEA were observed with higher trough plasma levels of marimastat, although this was not statistically significant.

Selection of a population of cancer patients with rapidly rising CEA levels during screening would tend to produce regression to the mean during the study period. This may contribute to the falls in the rate of rise of CEA occurring in patients treated at the two lowest doses (5 and 10 mg once daily). It seems reasonable to

**Table 5** Musculoskeletal events leading to dose reduction or treatment withdrawal

| Dose | *n* | No. continuing after day 28 | No. reducing dose or withdrawing | Time to event (days) |
|---|---|---|---|---|
| 5 mg once daily | 11 | 6 | 1 | 21 |
| 10 mg once daily | 12 | 1 | 0 | – |
| 20 mg once daily | 14 | 9 | 6 | 28, 49, 70, 76, 120, 175 |
| 10 mg twice daily | 11 | 6 | 4 | 41, 66, 95, 115 |
| 25 mg twice daily | 11 | 3 | 0 | – |
| 50 mg twice daily | 11 | 6 | 7 | 5, 16, 39, 45, 56, 58, 101 |

© Cancer Research Campaign 1999 

conclude, however, that the more pronounced reductions observed in the four higher dose groups were the result of treatment. A treatment effect on the rate of rise of CEA can be subject to a variety of interpretations, including the possibility of biochemical modulation of CEA synthesis or cancer antigen shedding unrelated to tumour progression. This seems unlikely in view of the lack of effect of marimastat on CEA shedding in in vitro studies with colorectal cancer cells (data not shown) and the apparent dose-related reduction in LDH, an important marker of tumour activity (Schwartz, 1992; Fountzilas et al, 1996). Finally, as a non-randomized study, patients with different characteristics could have been unevenly distributed across the marimastat doses, favouring the higher dose groups. Demographic data do not support this suggestion, although unmeasured variables may exist with the potential to confound results.

Marimastat was well-tolerated in this study, with musculoskeletal events emerging as the principal treatment-related toxicity. Symptoms were more severe and developed more rapidly at the highest dose (50 mg twice daily), although were reversible. Other adverse events were encountered commonly, but in most instances reflected the nature of the underlying disease process rather than a drug effect. The four serious adverse events thought possibly related to marimastat also were thought at least as likely to be related to the underlying disease.

On the basis of the safety and tolerability data, and the effects on cancer antigens, a dose range of 20 mg once daily to 25 mg twice daily seems appropriate for longer term, randomized and controlled studies. These studies should aim to define both the efficacy of the drug in terms of hard clinical end points such as patient survival or 'response' in terms of disease-free survival, and the long-term tolerability of this novel agent.

### ACKNOWLEDGEMENT

This study was supported by British Biotech Pharmaceuticals, Oxford, UK.

### REFERENCES

Allen-Mersh TG, Kemeny N, Niedzwiecki D, Shurgot B and Daly JM (1987) Significance of a fall in serum CEA concentration in patients treated with cytotoxic chemotherapy for disseminated colorectal cancer. *Gut* **28**: 1625–1629

An Z, Wang X, Willmott N, Chander SK, Tickle S, Docherty AJ, Mountain A, Millican AT, Morphy R, Porter RJ, Epemolu RO, Kubota T, Moossa AR and Hoffman RM (1997) Conversion of highly malignant colon cancer from an aggressive to a controlled disease by oral administration of a metalloproteinase inhibitor. *Clin Exp Metastasis* **15**: 184–195

Beckett RP, Davidson AH, Drummond AH, Huxley P and Whittaker M (1996) Recent advances in matrix metalloproteinase research. *Drug Dev Today* **1**: 16–26

Brown PD and Giavazzi R (1995) Matrix metalloproteinase inhibition: a review of anti-tumour activity. *Ann Oncol* **6**: 967–974

Fountzilas G, Gossios K, Zisiadis A, Svarna E, Skarlos D and Pavlidis N (1996) Prognostic variables in patients with advanced colorectal cancer treated with fluorouracil and leucovorin-based chemotherapy. *Med Ped Oncol* **26**: 305–317

Gijbels K, Masure S, Carton H and Opdenakker G (1992) Gelatinase in cerebrospinal fluid of patients with multiple sclerosis and other inflammatory neurological disorders. *J Neuroimmunol* **41**: 29–34

Gold P and Freedman SO (1965) Demonstration of tumor-specific antigens in human colonic carcinomata by immunological tolerance and adsorption techniques. *J Exp Med* **121**: 439

Gordon JL, Drummond AH and Galloway WA (1993) Metalloproteinase inhibitors as therapeutics. *Clin Exp Rheum* **11**: S91–S94

Gray ST, Yun K, Motoori T and Kuys YM (1993) Interstitial collagenase gene expression in colonic neoplasia. *Am J Pathol* **143**: 663–671

Hida J, Yasutomi M, Shindoh K, Kitaoka M, Fujimoto K, Ieda S, Machidera N, Kubo R, Morikawa E, Inufus H, Watatani M and Okuno K (1996) Second-look operation for recurrent colorectal cancer based on carcinoembryonic antigen and imaging techniques. *Dis Colon Rectum* **39**: 74–79

Ishikawa T, Ichikawa Y, Mitsuhashi M, Momiyama N, Chishima T, Tanaka K, Yamaoka H, Miyazakic K, Nagashima Y, Akitaya T and Shimada H (1996) Matrilysin is associated with progression of colorectal tumor. *Cancer Lett* **107**: 5–10

Kleiner DJ and Stetler-Stevenson WG (1993) Structural biochemistry and activation of matrix metalloproteinases. *Curr Opinions Cell Biol* **5**: 891–897

Levy AT, Cioce V, Sobel ME, Garbisa S, Grigioni WF, Liotta LA and Stetler-Stevenson (1991) Increased expression of the $M_r$ 72,000 type IV collagenase in human colonic adenocarcinoma. *Cancer Res* **51**: 439–444

Liotta LA and Stetler-Stevenson WG (1990) Metalloproteinases and cancer invasion. *Semin Cancer Biol* **1**: 99–106

Matrisian LM (1990) Metalloproteinases and their inhibitors in matrix remodelling. *Trends Genet* **6**: 121–125

Millar AW, Brown PD, Moore J, Galloway WA, Cornish AG, Lenehan TJ and Lynch KP (1998) Results of single and repeat dose studies of the oral matrix metalloproteinase inhibitor marimastat in healthy male volunteers. *Br J Clin Pharmacol* **45**: 21–26

Minton JP, Hoehn JL, Gerber DM, Horsley JS, Connolly DP, Salwan F, Fletcher WS, Cruz Jr AB, Gatchell FG and Oviedo M (1985) Results of a 400-patient carcinoembryonic antigen second-look colorectal cancer study. *Cancer* **55**: 1284–1290

Nakayama T, Watanabe M, Teramoto T and Kitajima M (1997) Slope analysis of CA19-9 and CEA for predicting recurrence in colorectal cancer patients. *Anticancer Res* **17**: 1379–1382

Nemunaitis J, Poole C, Primrose J, Rosemurgy A, Malfetano J, Brown P, Berrington A, Cornish A, Lynch K, Rasmussen H, Kerr D, Cox D and Millar A (1998) Combined analysis of studies of the effects of the matrix metalloproteinase inhibitor marimastat on serum tumor markers in advanced cancer: selection of a biologically active and tolerable dose for longer-term studies. *Clin Cancer Res* **4**: 1101–1109

Newell KJ, Witty JP, Rodgers WH and Matrisan LM (1994) Expression and localisation of matrix-degrading metalloproteinases during colorectal tumorigenesis. *Mol Carcinog* **10**: 199–206

O'Byrne EM, Parker DT, Roberts ED, Goldberg RL, MacPherson LJ, Blancuzzi V, Wilson D, Singh HN, Ludewig R and Ganu VS (1995) Oral administration of a matrix metalloproteinase inhibitor, CGS 27023A, protects the cartilage proteoglycan matrix in a partial meniscectomy model of osteoarthritis in rabbits. *Inflammation Res* **44**: S117–S118

Quentmeier A, Schlag P, Smok M and Herfarth C (1990) Re-operation for recurrent colorectal cancer: the importance of early diagnosis for resectability and survival. *Eur J Surg Oncol* **16**: 319–325

Rosenberg GA (1995) Matrix metalloproteinases in brain injury. *J Neurotrauma* **12**: 833–842

Saarialho-Kere UK, Vaalamo M, Puolakkainen P, Airola K, Parks WC and Karjalainen-Lindsberg ML (1996) Enhanced expression of matrilysin, collagenase and stromelysin-1 in gastrointestinal ulcers. *Am J Pathol* **148**: 519–526

Schwartz MK (1992) Enzymes as prognostic markers and therapeutic indicators in patients with cancer. *Clin Chim Acta* **206**: 77–82

Sugarbaker PH, Zamcheck N and Moore FDK (1976) Assessment of serial carcinoembryonic antigen (CEA) in postoperative management of colon and rectal cancer. *Cancer* **38**: 2310–2315

Wang X, Fu X, Brown PD, Crimmin MJ and Hoffman RM (1994) Matrix metalloproteinase inhibitor BB-94 (batimastat) inhibits human colon tumour growth and spread in a patient-like orthotopic model in nude mice. *Cancer Res* **54**: 4726–4728

Ward U, Primrose JN, Finan PJ, Perren TJ, Selby P, Purves DA and Cooper EH (1993) The use of tumour markers CEA, CA-195 and CA-242 in evaluating the response to chemotherapy in patients with advanced colorectal cancer. *Br J Cancer* **67**: 1132–1135

Watson SA, Morris TM, Robinson G, Crimmin MJ, Brown PD and Hardcastle JD (1995) Inhibition of organ invasion by the matrix metalloproteinase inhibitor batimastat (BB-94) in two human colon carcinoma metastasis models. *Cancer Res* **55**: 3629–3633

Zeng SZ, Huang Y, Cohen AM and Guillem JG (1996) Prediction of colorectal cancer relapse and survival via tissue RNA levels of matrix metalloproteinase-9. *J Clin Oncol* **14**: 3133–3140

Zucker S, Lysik RM, Zarrabi MH and Moll U (1993) $M_r$ 92,000 type IV collagenase is increased in plasma of patients with colon cancer and breast cancer. *Cancer Res* **53**: 140–146

 *© Cancer Research Campaign 1999*
